# C1q/Tumor Necrosis Factor-Related Protein-9 Enhances Macrophage Cholesterol Efflux and Improves Reverse Cholesterol Transport via AMPK Activation

**DOI:** 10.1007/s10528-024-10761-1

**Published:** 2024-04-10

**Authors:** Xiaosu Song, Gaizhen Liu, Yunfei Bin, Rui Bai, Bin Liang, Huiyu Yang

**Affiliations:** Department of Cardiology, The Second Hospital of Shanxi Medical University, 382 Wuyi Road, Taiyuan, China

**Keywords:** AMPK, Atherosclerosis, CTRP9, Reverse cholesterol transport

## Abstract

Cholesterol efflux from foam cells in atherosclerotic plaques is crucial for reverse cholesterol transport (RCT), an important antiatherogenic event. ATP-binding cassette (ABC) transporters, ABCA1 and ABCG1, are key receptors in the cholesterol efflux pathway. C1q/tumor necrosis factor-related protein-9 (CTRP9) is a newly discovered adipokine and exhibits an atheroprotective activity. However, the role of CTRP9 in RCT still remains unknown. In this work, we investigated the effect of subcutaneous administration of CTRP9 protein on RCT and atherosclerotic lesion formation in *ApoE*^−/−^ mice fed with a high-fat diet. CTRP9-dependent regulation of cholesterol efflux and ABC transporters in RAW 264.7 foam cells was determined. Our results showed that CTRP9 protein decreased atherosclerotic lesions, increased cholesterol efflux, and upregulated liver ABCA1 and ABCG1 expression in *ApoE*^−/−^ mice. CTRP9 treatment dose-dependently increased mRNA and protein expression of ABCA1, ABCG1, and LXR-α in RAW 264.7 foam cells. Moreover, the expression and phosphorylation of AMPK was potentiated upon CTRP9 treatment. Notably, CTRP9-induced cholesterol efflux and upregulation of ABCA, ABCG1, and LXR-α were impaired when AMPK was knocked down. AMPK depletion restored cholesterol accumulation in CTRP9-treated RAW 264.7 cells. Taken together, subcutaneous injection is an effective novel delivery route for CTRP9 protein, and exogenous CTRP9 can facilitate cholesterol efflux and promote RCT in an animal model of atherosclerosis. The atheroprotective activity of CTRP9 is mediated through the activation of AMPK signaling.

**Supplementary Information:**

The online version contains supplementary material available at 10.1007/s10528-024-10761-1.

## Introduction

Atherosclerosis is regarded as a pathological factor for various cardiovascular diseases such as coronary heart disease, stroke, and peripheral vascular disorder. The formation of foam cells is a hallmark of early atherosclerotic plaques (Yu et al. 2013). Multiple modulators are involved in the complicated process of atherosclerotic lesion formation. ATP-binding cassette (ABC) transporters, especially ABCA1 and ABCG1, have been shown to play important roles in the regulation of cholesterol homeostasis (Dean et al. 2001; Tarling and Edwards 2011). They mediate the efflux of cholesterol and phospholipids to lipid-poor apolipoproteins, a key event of reverse cholesterol transport (RCT) (Ouimet et al. 2019). Liver-X-receptor (LXR) is cholesterol-sensing nuclear receptor. The LXR pathway regulates lipid metabolism and transport (Wang and Tontonoz 2018). LXR consists of two subunits, LXR-α and LXR-β, which are encoded by two independent genes. Activation of LXR-α can upregulate the expression of ABCA1, ABCG1, and SR-BI, thus promoting macrophage cholesterol efflux (Bonamassa and Moschetta 2013). These transporters and receptors involved in RCT are potential therapeutic targets for atherosclerosis.

It has been reported that adipose tissue can release multiple bioactive adipokines, such as leptin, omentin, adiponectin, and C1q/tumor necrosis factor-related proteins (CTRPs) (Shibata et al. 2014; Fang and Judd 2018; Maruyama et al. 2012; Yu et al. 2018). These adipokines regulate glucose and lipid metabolism and elicit anti-inflammatory and anti-atherosclerotic effects. Recently, compelling studies have indicated that CTRP9, a newly identified paralog of adiponectin (Wong et al. 2009), plays a protective role in atherosclerosis. In patients with coronary atherosclerosis disease (CAD), serum CTRP9 level is significantly decreased (Sun et al. 2013). Similar results are observed in a mouse model of acute myocardial infarction (Wang et al. 2015). CTRP9 exerts its atheroprotective effects through inhibition of vascular inflammation (Jung et al. 2016), alteration of lipid metabolism (Yang et al. 2016), amelioration of endothelial dysfunction (Zheng et al. 2011; Sun et al. 2017), and regulation of vascular smooth muscle cell (VSMC) phenotype (Liu et al. 2017). However, the role of CTRP9 in RCT remains unclear.

Overexpression of mouse *CTRP9* gene via a lentiviral expression system can prevent the development of atherosclerotic lesions in *ApoE*^−/−^ mice fed with HFD (Huang et al. 2019). In the present study, we investigated the robust effect of subcutaneous injection of CTRP9 protein on atherosclerotic lesion formation and cholesterol efflux. Additionally, we investigated the mechanisms responsible for CTRP9-mediated atheroprotection.

## Materials and Methods

### Animal Models

Our study was conformed with the US National Institutes of Health Guide for the Care and Use of Laboratory Animals and approved by the Institutional Animal Care and Use Committee of Shanxi Medical University (Taiyuan, China).

Eight-week-old male *ApoE*^−/−^ mice were purchased from Laboratory Animal Center of Peking University (Beijing, China). All mice were maintained on a high-fat diet (21% [wt/wt] fat, 0.21% cholesterol) during the whole experimental period. Mice were randomized into control and CTRP9 groups. Ten weeks after feeding the high-fat diet, mice received vehicle or human CTRP9 (0.25 μg/g/day; Aviscera Bioscience, Santa Clara, CA, USA; catalog number: 00081-04-100) for additional 4 weeks via mini-osmotic minipumps (DURECT Corporation, Cupertino, CA, USA), which were inserted subcutaneously into the back of the mice.

### In Vivo Macrophage RCT Assay

In vivo RCT assay was performed as previously described (Liang et al. 2017; Zhang et al. 2003). Ox-LDL (catalog number: 20605ES05; Yeasen Biotechnology, Shanghai, China) at a final concentration of 50 µg/ml was radiolabeled with [^3^H] cholesterol (5 µCi/ml). The mixture was bathed in water at 37 °C for 30 min, added into RAW 264.7 cell cultures and cultured for another 48 h. Cells were resuspended after centrifugation, and the count was adjusted to 10.0 × 10^9^ cells/L. Radiolabeled cells (500 μl) with the radioactive activity of 6.2 × 10^6^ cpm/ml were intraperitoneally injected into each mouse. Forty-eight hours later, the mice were anesthetized using 1% pentobarbital sodium at a dose of 50 mg/kg. Blood samples were obtained from retro-orbital plexus. After centrifuging for 5 min at 1200 r/min, 20 μL of plasma was extracted and used for liquid scintillation counting. Feces were homogenized in 50% ethyl alcohol overnight and subjected to liquid scintillation counting. The mice were sacrificed, and liver samples were collected for liquid scintillation counting.

### Plasma Lipid Profile Analysis

Blood samples of mice were collected using tubes containing EDTA (2.0 mg/ml). After centrifuging at 2000×*g* for 15 min at 4 °C, plasma was separated. Triglyceride (TG; catalog number: BC0625), total cholesterol (TC; catalog number: BC1985), LDL-C (catalog number: BC5335), and HDL-C (catalog number: BC5325) were determined by enzymatic kits (Solarbio, Beijing, China) according to the manufacturer’s instructions.

### Evaluation of Aortic Lesions

Aortic roots were dissected and fixed as previously described (Liang et al. 2017). Hearts were cut directly under and parallel to the leaflet, and the upper portions were embedded in OCT medium and kept at − 24 °C. The aortic sinus was cut into sections of 8 mm in thickness. Twenty sections were stained with Oil-red O (catalog number: O8010; Solarbio) for lipids. Lesion areas were determined by ImageJ software (National Institutes of Health, America). Data were recorded as lesion size ± SEM.

### Cell Culture and Treatments

RAW 264.7 cells (Cell Bank of Chinese Academy of Sciences, Shanghai, China) were plated on 6-well plates in Roswell Park Memorial Institute (RPMI) 1640 medium supplemented with 10% FBS and 2% penicillin–streptomycin and cultured at 37 °C (5% CO_2_). Upon reaching 85% confluence, cells were transformed into foam cells by incubation with 50 μg/mL ox-LDL for 24 h. Foam cells were then treated with 0.3, 1, and 3 μg/ml CTRP9 for 3 h.

### Small Interfering RNA (siRNA) Transfection

siRNA duplex oligonucleotides were designed for human cDNAs encoding AMP kinase-α1 (AMPK-α1) (GenePharma, Shanghai, China). The siRNA sequences were 5′-UGCCUACCAUCUCAUAAUAdTdT-3′ (sense) and 5′-UAUUAUGAGAUGGUAGGCAdT dT-3′ (antisense). After reaching 80% confluence, RAW 264.7-derived foam cells were transfected with AMPK-siRNA or negative control siRNAs at a final concentration of 50 nM using Lipofectamine RNAiMAX (Invitrogen, Carlsbad, CA, USA; catalog number: 13778150).

### RNA Isolation and Real-Time Quantitative PCR

Animals were anesthetized with pentobarbital and livers were collected. Total RNA from tissues and cells was extracted using Trizol reagent according to the manufacturer’s protocol. The targeted genes and primer sequences are as follows: *ABCA1* forward: 5′-AACAGTT TGTGGCCCTTTTG-3′, reverse: 5′-AGTTCCAGGCTGGGGTACTT-3′, *ABCG1* forward: 5′-GTGACGCTGACTATAAGAGA-3′, reverse: 5′-AGGTGATTCGCAGATGTG-3′, *GAPDH* forward: 5′-CGCTCTCTGCTCCTCCTGTT-3′, reverse: 5′-CCATGGTGTCTGAGCGATGT-3′, *LXR-α* forward: 5′-CTACAACCACGAGACAGAA-3′, reverse: 5′-GGCGATAAGCAAGGCATA-3′, AMPK forward: 5′-UGCCUACCAUCUCAUAAUAdTdT-3′, reverse: 5′-UAUUAUGAGAUGGUAGGCAdTdT-3′. Real-time quantitative PCR using SYBR green was performed in an ABI 7300 real-time PCR system (Applied Biosystems, Foster City, CA, USA). Samples were subjected to 95 °C denaturation for 2 min, followed by 40 cycles of 95 °C for 5 s and 60 °C for 30 s. Relative fold changes were determined using the formula 2^−ΔΔCt^.

### Western Blotting Analysis

Protein was extracted from liver tissues and RAW 264.7-derived foam cells using lysis buffer containing protease inhibitors (catalog number: P8340; Sigma-Aldrich, St. Louis, MO, USA). Protein samples (30 μg) were separated on a 10% SDS-PAGE gel and transferred to PVDF membranes. Nonspecific proteins were blocked by 5% skim milk in Tris buffered saline containing 0.1% Tween 20 for 1 h at room temperature. Membranes were incubated overnight with primary antibodies to ABCA1 (1:300; catalog number: ab307536), ABCG1 (1:500; catalog number: ab52617), AMPK (1:1000; catalog number: ab32047), LXRα (1:1000; catalog number: ab41902), or GAPDH (1:2000; catalog number: ab8245) (Abcam, Cambridge, MA, USA), and then with secondary antibodies (catalog number: ab270144 or catalog number: ab205719, Abcam) for 1 h. Images were captured and quantified using a Bio-Rad ChemiDoc XRS system. Immunoreactive protein bands were detected with ECL test. The relative protein levels were expressed as the ratio of protein to GAPDH densitometric values.

### Cholesterol Efflux Assay

Cellular cholesterol efflux was measured as previously described (Liang et al. 2017). Macrophages were cultured and labeled with 0.2 μCi/mL [^3^H] cholesterol for 72 h. After washing, equilibrated [^3^H] cholesterol-labeled cells were incubated in efflux medium containing RPMI 1640 medium and 0.1% bovine serum albumin (BSA) with human plasma ApoA-I (20 μg/mL) for 6 h. Monolayers were lysed, and cellular lipids were extracted with isopropanol. Concentration of [^3^H] cholesterol in medium and cells was measured by liquid scintillation counting. The following formula was used to determine the cholesterol efflux: Cholesterol efflux (%) = [total media counts / (total cellular counts + total media counts)] × 100.

### High-Performance Liquid Chromatography (HPLC)

HPLC analysis was conducted as previously described (Liang et al. 2017). Cells were collected and lysed fully by sonication. A 0.1-mL aliquot of cell solution (containing 5–20 μg protein) was used to measure the free cholesterol (FC), and another aliquot was used to detect total cholesterol (TC). A 15% KOH ethanol solution (50 μL) was prepared and heated for 2 h to allow for TC extraction. 300 μL ethanol solution was prepared for FC extraction. Then 500 μL N-hexane:isopropanol (4:1) was added and stirred for 1 min at room temperature. After centrifugation at 3600×*g* for 5 min, supernatant was collected and frozen in liquid nitrogen. 20 μL of dried samples was reconstituted into mobile phase (isopropanol:acetonitrile 50:50) and injected into the HPLC system (Waters Corporation, Milford, MA, USA). Analysis was performed using a C8 reversed-phase ODS2 HPLC column. Data were analyzed using Empower software (Waters Corporation).

### Statistical Analysis

All data are presented as means ± standard deviation. Statistical comparisons were done by Student’s *t* test or one-way analysis of variance (ANOVA) with a post hoc multiple-comparison test. All statistical analyses were conducted using SPSS 22.0 software (SPSS, Chicago, IL, USA). *P* value less than 0.05 was considered as statistically significant.

## Results

### CTRP9 Decreases Lesion Area of Aortic Sinus Sections in ApoE^−/−^ Mice Fed with HFD

**Fig. 1:**
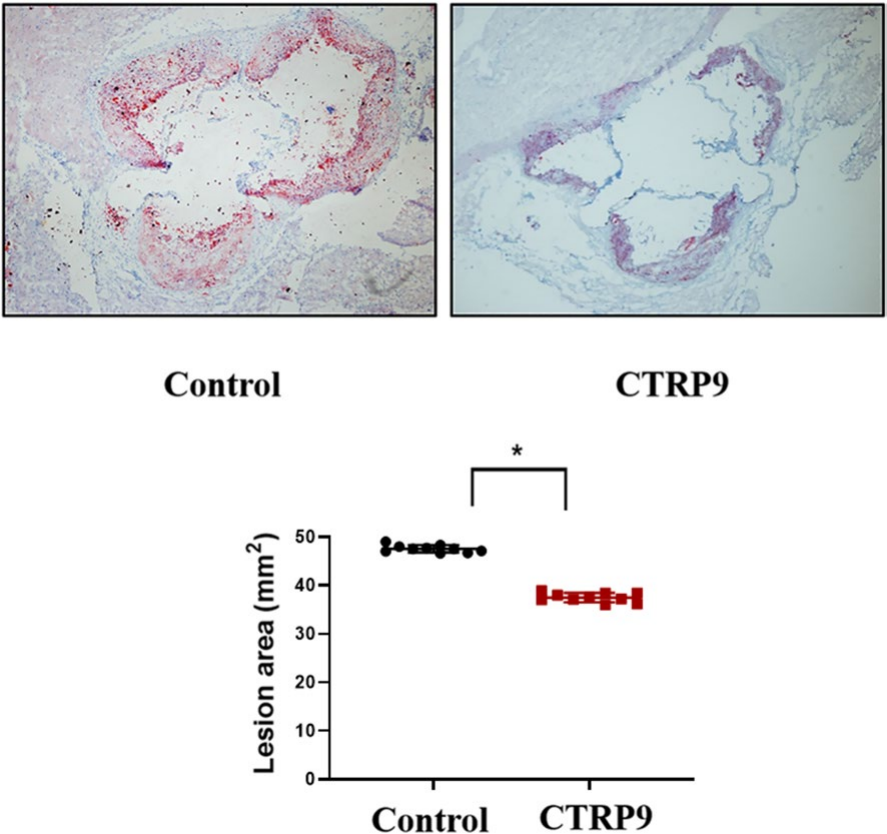
CTRP9 decreases aortic sinus lesion area size in *ApoE*^−/−^ mice fed with HFD. Representative staining of aortic sinus with Oil Red O in *ApoE*^−/−^ mice fed with HFD for 10 weeks. Values were expressed as mean ± standard error of the mean (*n* = 10 per group). *P < 0.05 *vs*. control group

We first investigated the effect of CTRP9 on the formation of atherosclerotic plaques in *ApoE*^−/−^ mice fed with a high-fat diet. After 4-week administration of CTRP9, quantification of lipid accumulation in aortic sinus sections by Oil Red O staining revealed that CTRP9 significantly decreased lesion areas by 10.43% in *ApoE*^−/−^ mice, compared with control group (Fig. 1). These data suggest that CTRP9 can attenuate the development of atherosclerosis.

### CTRP9 Increases Cholesterol Efflux and Affects Plasma Lipid In Vivo

**Fig. 2:**
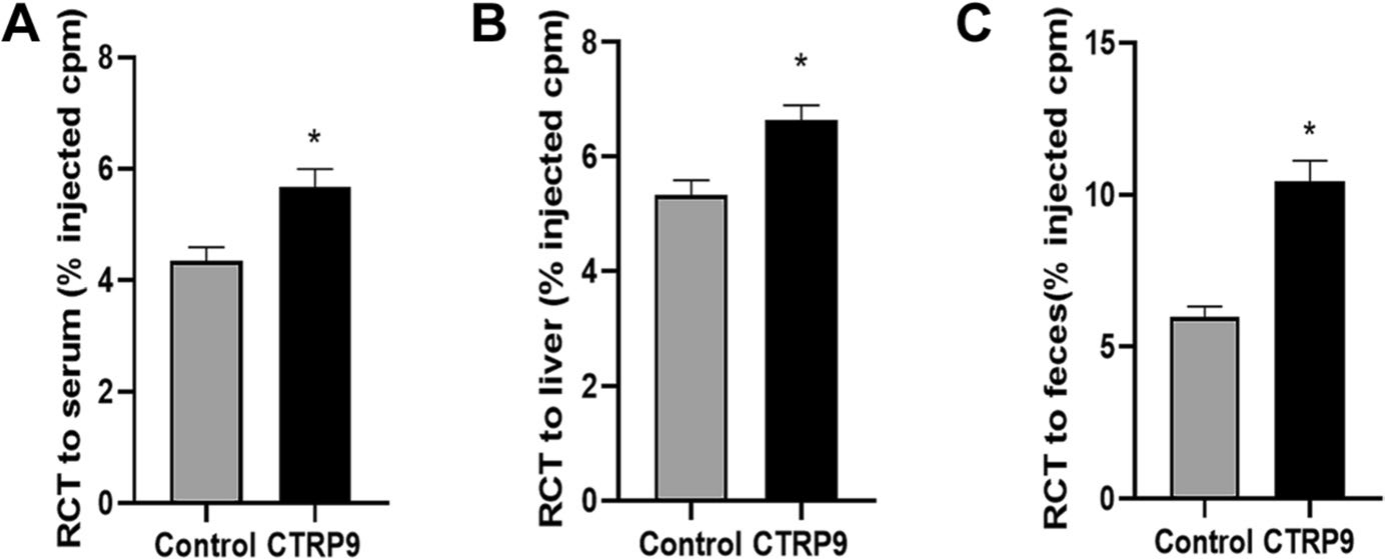
CTRP9 increases reverse cholesterol transport in vivo. 3H-cholesterol radiolabeled ox-LDL-loaded macrophages were injected to each group. Blood, liver, and feces were collected after 48 h for the analysis of RCT. Data were expressed as the ratio of 3H-cholesterol tracer to total cpm tracer injected. **A** Percentage of plasma 3H-cholesterol tracer. **B** Percentage of hepatic 3H-cholesterol tracer. **C** Percentage of fecal 3H-cholesterol tracer. Values were expressed as mean ± standard deviation (*n* = 6 per group). *P < 0.05 *vs*. control group

To further assess the effects of CTRP9 on RCT in vivo, [^3^H] cholesterol radiolabeled RAW 264.7 macrophages were intraperitoneally injected to *ApoE*^−/−^ mice. Radioactivity of [^3^H] cholesterol was determined by liquid scintillation counting. The results showed that the radioactivities of plasma, liver, and feces samples were increased after the treatment with CTRP9 (Fig. 2).

Next, we determined plasma lipid, including TG, TC, LDL-C, and HDL-C levels in each group. Our results indicated that CTRP9 upregulated plasma HDL-C levels and downregulated plasma TC and LDL-C levels, compared with control (Table 1). No difference in the TG levels between the CTRP9 and control groups was observed.

**Table 1:** CTRP9 regulates plasma lipid profile in *ApoE* ^−/−^ mice fed with HFD

	Body Weight (g)	TG (mg/dL)	TC (mg/dL)	LDL-C (mg/dL)	HDL-C (mg/dL)
Control	30.1 ± 1.3	115 ± 10	412 ± 12	340 ± 12	58 ± 6
CTRP9 (0.25 g/g/day)	29.5 ± 0.9	112 ± 4	342 ± 15^*^	297 ± 15^*^	70 ± 3^*^

Plasma lipid profile was measured using enzymatic method for each group

Values were expressed as mean ± standard deviation (*n* = 10 per group), *P < 0.05 *vs*. control group

### CTRP9 Upregulates ABCA1, ABCG1, and LXR-α Expression in ApoE^−/−^ Mice Fed with HFD

**Fig. 3:**
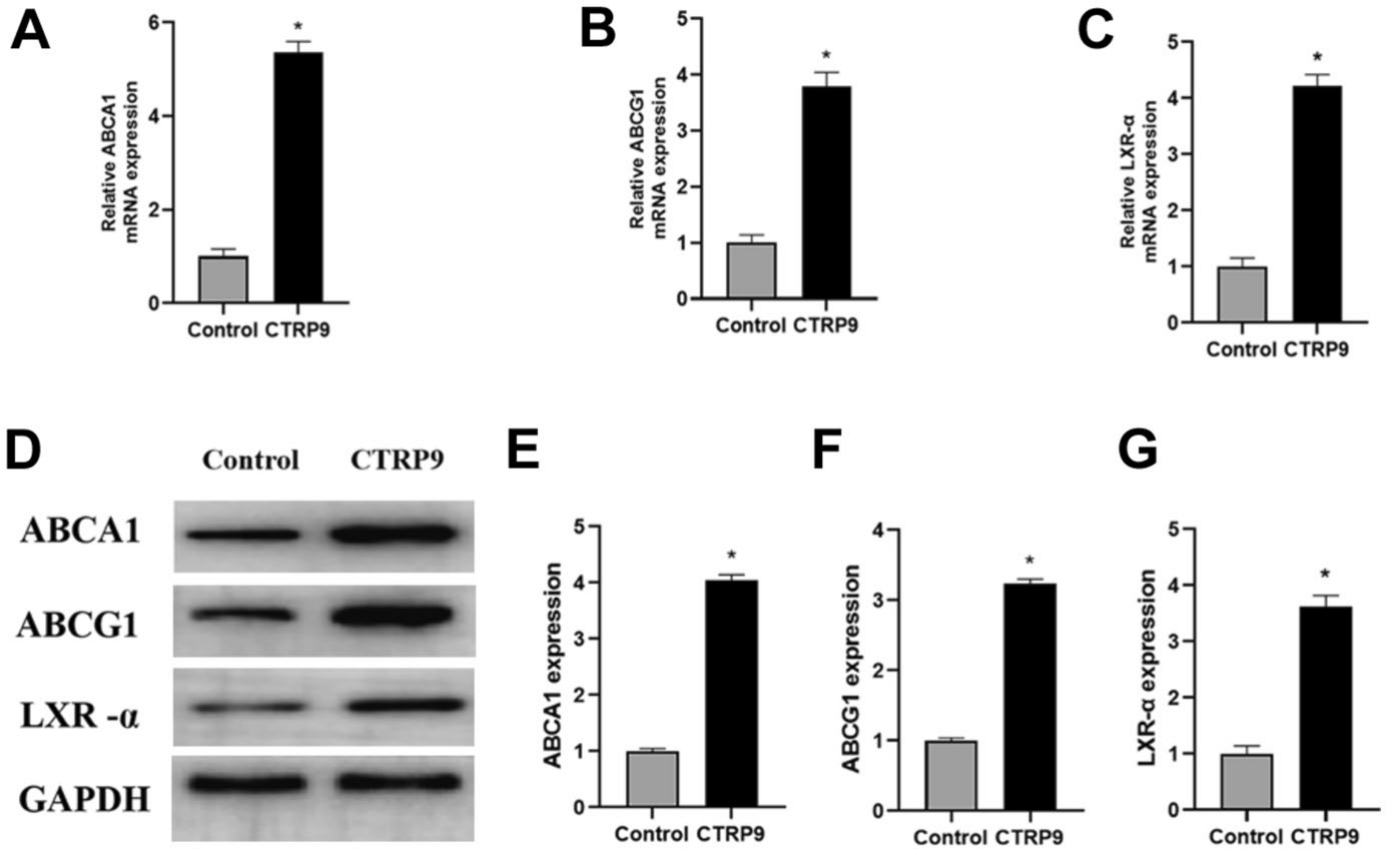
CTRP9 upregulates ABCA1, ABCG1, and LXR-α expression in livers of *Apo E*^−/−^ mice fed with HFD. **A, B, C** Expression of ABCA1, ABCG1, and LXR-α mRNA was quantified by real-time quantitative PCR. **D, E, F, G** Protein levels were measured by Western blotting. Data were shown as the mean ± standard deviation from three independent experiments. *P < 0.05 *vs*. control group

In the progression of atherosclerosis, ABCA1 and ABCG1 play critical roles in mediating cholesterol efflux from macrophages to HDL. ABCA1 can assemble effluxed cellular cholesterol with apolipoprotein A-I (ApoA-I) to form nascent HDL. To further evaluate the impact of CTRP9 on the regulation of cholesterol efflux, we measured the mRNA expression of ABCA1 and ABCG1 in livers of *ApoE*^−/−^ mice fed with HFD. As shown in Fig. 3A and B CTRP9 significantly increased the mRNA levels of ABCA1 and ABCG1 relative to the control group. Consistent results were observed when we analyzed the protein levels of ABCA1 and ABCG1 (Fig. 3D–F).

LXR-α is capable of stimulating the transcription of ABCA1 and ABCG1 (Costet et al. 2000). As demonstrated above, CTRP9 regulated the expression of ABCA1 and ABCG1 in RAW 264.7 macrophages. To further confirm whether CTRP9 has an impact on the expression of LXR-α, we performed real-time quantitative PCR and Western blot analyses to measure the mRNA (Fig. 3C) and protein level (Fig. 3D and G) of LXR-α. The results showed that administration of CTRP9 significantly induced the expression of LXR-α mRNA and protein in vivo.

### CTRP9 Decreases Cholesterol Content and Increases Cholesterol Efflux to ApoA-I in RAW 264.7 Macrophage-Derived Foam Cells

**Fig. 4:**
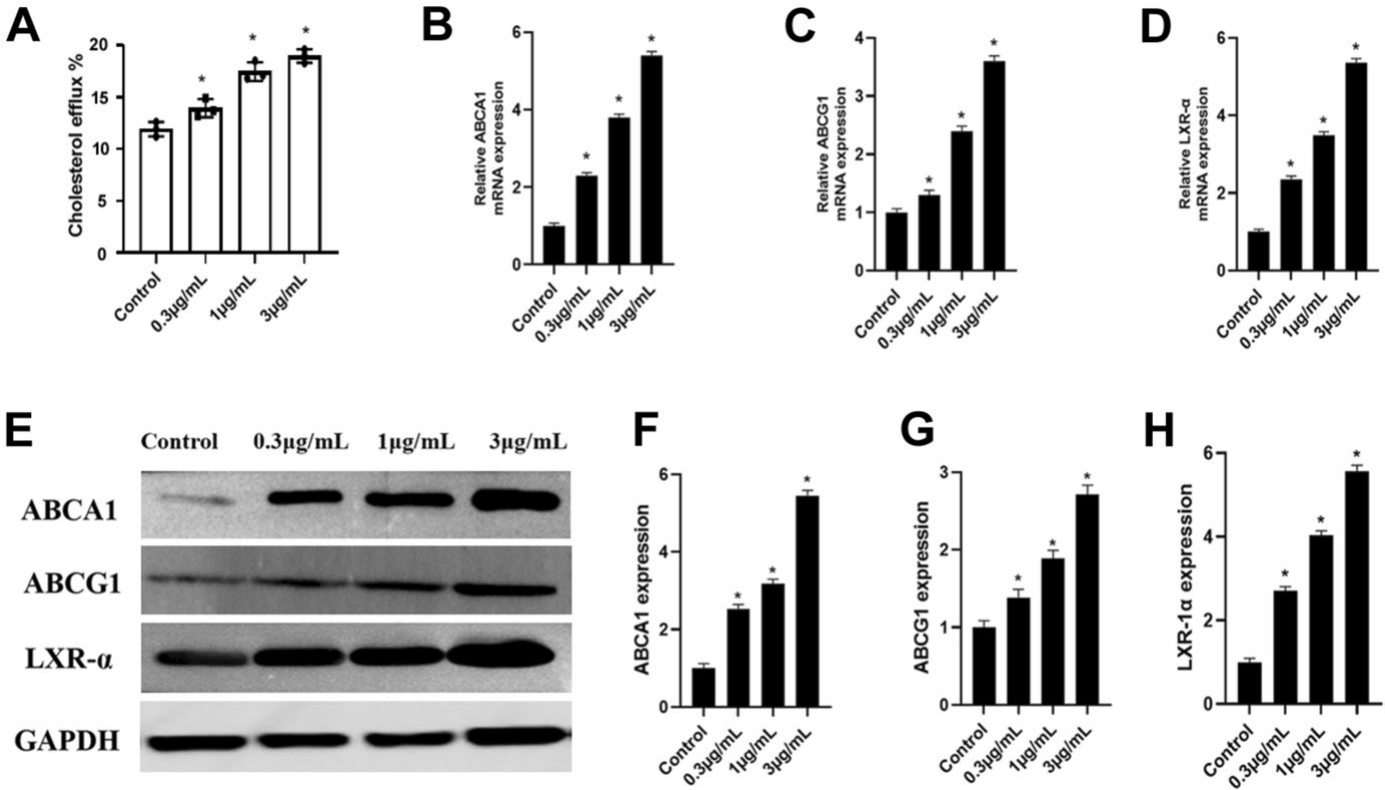
CTRP9 upregulates cholesterol efflux and expression of ABCA1, ABCG1, and LXR-α in RAW 264.7 macrophage-derived foam cells. **A** ApoA-I-mediated cholesterol efflux in RAW 264.7 macrophage-derived foam cells was analyzed by liquid scintillation counting assays. **B, C, D** Expression of ABCA1, ABCG1, and LXR-α mRNA was quantified by real-time quantitative PCR. **E, F, G, H** Protein levels were measured by Western blotting. Data were shown as mean ± standard deviation from three independent experiments. *P < 0.05 *vs*. control group

To further elucidate the mechanism of CTRP9 on cholesterol regulation, we treated foam cells derived from RAW 264.7 macrophages with different concentration of CTRP9. The results showed that CTRP9 can reduce cellular TC (total cholesterol), FC (free cholesterol), and CE (cholesterol ester) in a dose-dependent way (Table 2). Next, we measured the cholesterol efflux to ApoA-I in each group by liquid scintillation counting. As shown in Fig. 4A, administration of CTRP9 increased cholesterol efflux to ApoA-I in a dose-dependent manner.

**Table 2:** CTRP9 regulates cholesterol content in RAW 264.7 macrophage-derived foam cells

	TC (mg/dL)	FC (mg/dL)	CE (mg/dL)	CE/TC (%)
Control	426 ± 40	168 ± 17	258 ± 21	60.1
0.3 μg/mL CTRP9	410 ± 25	165 ± 14	245 ± 20	59.7
1 μg/mL CTRP9	297 ± 17^*^	123 ± 12^*^	174 ± 11^*^	58.6
3 μg/mL CTRP9	213 ± 11^*^	90 ± 11^*^	123 ± 17^*^	57.7

Foam cells derived from RAW 264.7 macrophages were treated with 0.3 μg/mL, 1 μg/mL, and 3 μg/mL CTRP9 for 3 h, and HPLC was performed to measure the levels of cellular TC, CE, and FC

Data were shown as mean ± standard deviation from three independent experiments

*P < 0.05 *vs*. control group

### CTRP9 Upregulates ABCA1, ABCG1, and LXR-α Expression in RAW 264.7 Macrophages-Derived Foam Cells

Given that cholesterol efflux was increased by CTRP9, we further investigated the expression of ABCA1 and ABCG1 by real-time quantitative PCR and Western blot analyses. Consistent with what we observed in vivo, CTPR9 treatment increased the expression of ABCA1, ABCG1, and LXR-α at the mRNA (Fig. 4B and C) and protein (Fig. 4E–G) levels in RAW 264.7macrophages-derived foam cells. Such increase was in a dose-dependent manner.

### CTRP9 Upregulates AMPK Expression in RAW 264.7 Foam Cells

**Fig. 5:**
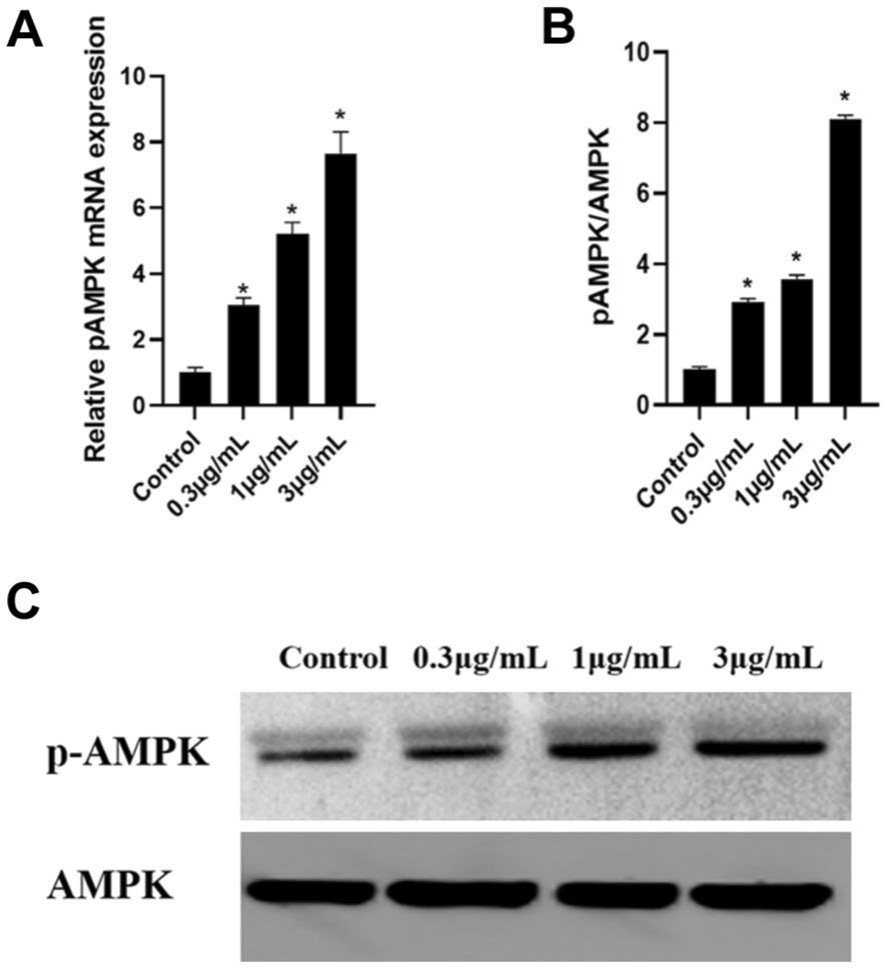
CTRP9 upregulates AMPK phosphorylation in RAW 264.7 macrophage-derived foam cells. **A** Phosphorylation of AMPK mRNA was quantified by real-time quantitative PCR. **B, C** Protein levels were measured by Western blot. Data were shown as the mean ± standard deviation from three independent experiments. *P < 0.05 *vs*. control group

AMPK regulates cellular energy metabolism and acts downstream of CTRP9 signaling (Sun et al. 2017; Kambara et al. 2015). We next assessed the effect of CTRP9 on the expression and activation of AMPK. The results showed that CTRP9 stimulated the mRNA expression of AMPK in RAW 264.7 foam cells (Fig. 5A). Moreover, the phosphorylation of AMPK protein was enhanced upon CTRP9 treatment (Fig. 5B and C).

### AMPK is Involved in the CTRP9-Mediated Regulation of ABCA1 and ABCG1 and Cholesterol Efflux

**Fig. 6:**
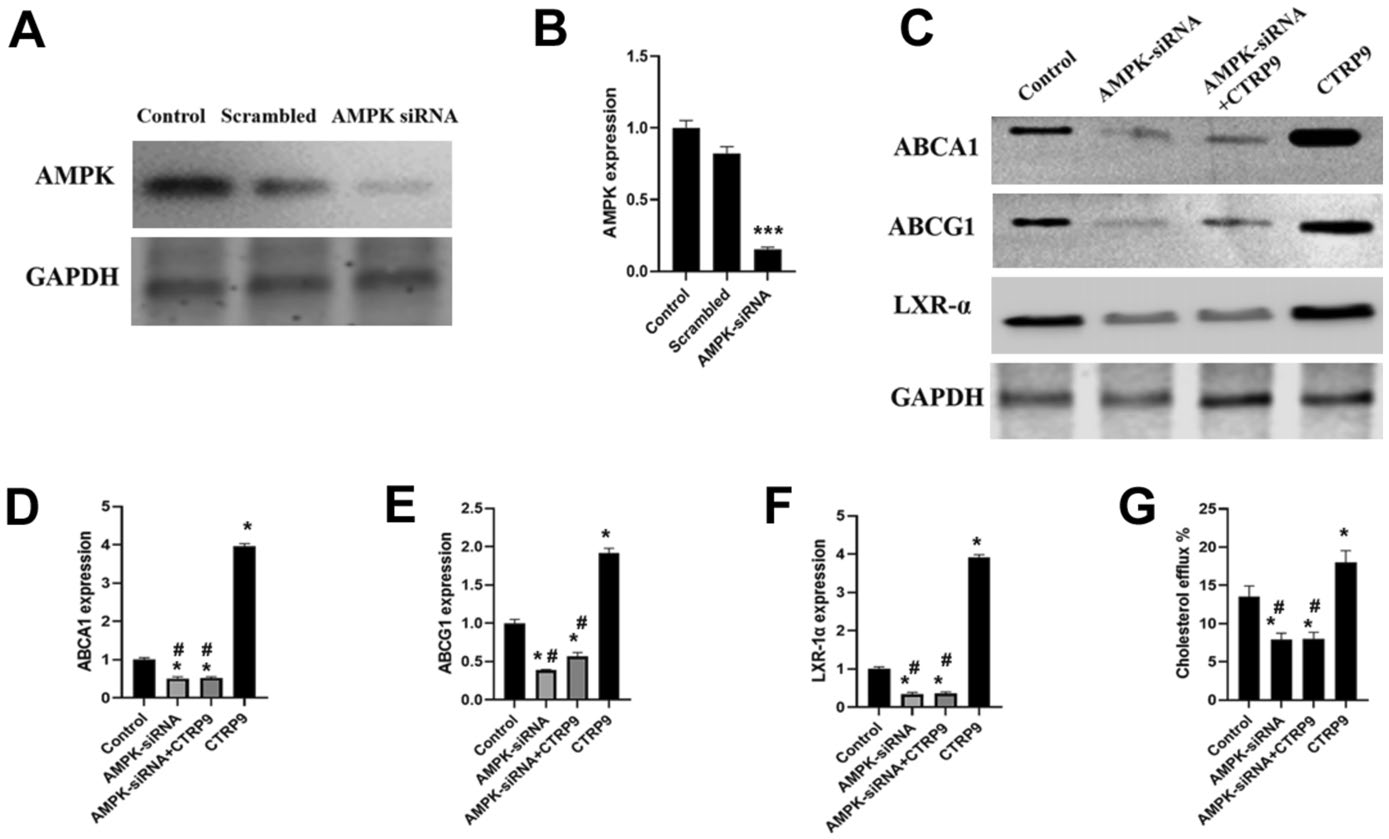
AMPK activation is involved in CTRP9 upregulation of ABCA1, ABCG1, LXR-α, and cholesterol efflux. **A, B** Knockdown of AMPK by siRNA was confirmed using Western blot analysis (> 80% knockdown). **C, D, E, F** AMPK knockdown blocked CTRP9-mediated upregulation of ABCA1, ABCG1, and LXR-α expression. **G** Cellular cholesterol efflux from RAW 264.7 macrophages was analyzed using liquid scintillation counting assays. Data were shown as mean ± standard deviation from three independent experiments. *P < 0.05 *vs*. control group, ***P < 0.001 *vs*. control group, ^#^*P* < 0.05 *vs*. CTRP9 group

To test whether CTRP9 increases ABCA1 and ABCG1 expression by activating the APMK pathway, we knocked down AMPK using siRNAs (> 85%, Fig. 6A and B). When treated with AMPK-siRNAs, the upregulation of ABCA, ABCG1, and LXR-α by 3 μg/mL of CTRP9 was completely suppressed (Fig. 6C–F). Moreover, the cholesterol efflux to ApoA-I was suppressed when AMPK was depleted (Fig. 6G). Intracellular cholesterol content was increased in AMPK-depleted cells (Table 3). Taken together, these results suggested that the effect of CTRP9 on cholesterol efflux is dependent on the AMPK signaling pathway.

**Table 3:** CTRP9 regulates cholesterol efflux in an AMPK independent manner

	TC (mg/dL)	FC (mg/dL)	CE (mg/dL)	CE/TC (%)
Control	416 ± 35	167 ± 15	249 ± 23	59.9
AMPK-siRNA	610 ± 25^*^	236 ± 14^*^	374 ± 22^*^	61.3
AMPK-siRNA + CTRP9	627 ± 28^*#^	243 ± 16^*#^	384 ± 14^*#^	61.2
CTRP9	215 ± 17^*^	92 ± 11^*^	123 ± 15^*^	57.2

RAW 264.7 macrophages-derived foam cells were transfected with control or AMPK-siRNA and then incubated with 3 μg/mL CTRP9 for 3 h. HPLC was performed to measure the levels of cellular TC, CE, and FC

Data were shown as mean ± standard deviation from three independent experiments

*P < 0.05 vs. control group, ^#^*P* < 0.05 vs. CTRP9 group

## Discussion

In this work, we suggest a novel delivery strategy and show that subcutaneous administration of CTRP9 yields atheroprotective effects in the *ApoE*^−/−^ mouse model. CTRP9 attenuates atherosclerotic lesions and promotes cholesterol efflux in *ApoE*^−/−^ mice. Previous studies have indicated the anti-inflammatory activity of CTRP9 in RAW 264.7 macrophages (Zhang et al. 2019). Our data provide direct evidence for the regulation of cholesterol efflux by CTRP9 in RAW 264.7 cells. Moreover, activation of AMPK signaling is responsible for CTRP9-dependent regulation of cholesterol efflux. Our results collectively point toward the potential of exogenous CTRP9 in preventing atherosclerosis.

As the closest paralog of adiponectin in CTRP family, CTRP9 is associated with atherosclerosis. In CAD patients, serum CTRP9 levels are negatively correlated with TNF-α and IL-6 levels, while positively correlated with high-density lipoprotein (HDL-C) levels (Wang et al. 2015). Decreased CTRP9 levels are an independent risk factor for thin fibrous cap atherosclerosis in patients with coronary heart disease (Liu et al. 2022). These studies suggest the contribution of CTRP9 to the occurrence of atherosclerosis. ApoE plays a crucial role in the removal of cholesterol-rich lipoproteins from the plasma due to its high affinity binding to low-density lipoprotein (LDL) receptor on the surface of hepatocytes. Its loss leads to hypercholesterolemia and atherosclerosis in mice fed with the HFD (Alagarsamy et al. 2022). The *ApoE*^−/−^ mouse model has been commonly used to recapitulate the disrupted lipid metabolism and atherosclerotic development in humans. Hence, we utilized the *ApoE*^−/−^ mouse model to evaluate the effect of exogenous CTRP9 on atherosclerosis. Lentiviral delivery of mouse CTRP9 can prevent the development of atherosclerotic lesions in *ApoE*^−/−^ mice fed with HFD (Huang et al. 2019). Here, we delivered CTRP9 protein through a different route and demonstrated that subcutaneous administration of human CTRP9 attenuates HFD-induced atherosclerotic plaque formation in *ApoE*^−/−^ mice.

Multiple studies have demonstrated that CTRP9 is antiatherogenic through various mechanisms. It has been reported that CTRP9 inhibits the uptake of cholesterol by VSMCs and protects VSMCs from cholesterol-induced damage (Liu et al. 2017). CTRP9 overexpression in *ApoE* knockout mice exhibit a significant reduction of lipid contents in carotid plaques (Huang et al. 2019). These studies indicate a protective role for CTRP9 in regulating lipid metabolism. Our in vivo study reveals that CTRP9 administration influences plasma lipid levels, especially cholesterol contents. The administration of CTRP9 significantly increases the serum level of HDL-C and decreases the serum levels of TC and LDL-C. Moreover, we show that CTRP9 significantly augments cholesterol efflux to serum, livers, and feces. CTRP9-mediated enhancement of cholesterol efflux is also observed in foam cells and VSMCs (Liu et al. 2017; Lei et al. 2021). These results suggest that exogenous CTRP9 improves lipid metabolism and thus ameliorates HFD-induced atherosclerotic plaque formation in *ApoE*^−/−^ mice.

ATP-binding cassette (ABC) transporters ABCA1 and ABCG1 play crucial and complementary roles in mediating cholesterol efflux (Phillips 2014). While ABCA1 transfers cholesterol to lipid-free apolipoproteins, such as ApoA-I and ApoE, for the neogenesis of HDL in the early steps of cholesterol efflux from peripheral cells (Hong and Tontonoz 2014), ABCG1 transfers cholesterol from macrophage to large HDL particles (Westerterp et al. 2014). In atherosclerosis, the expression of ABCA1 and ABCG1 is decreased, which aggravates the accumulation of cholesterol and formation of foam cells. So, these two ABC transporters are key targets to regulate RCT in the progression of atherosclerosis. In our in vivo studies, we demonstrated that CTRP9 can upregulate ABCA1 and ABCG1 expression both in mRNA and in protein levels compared with control group in *ApoE*^−/−^ mice fed with HFD. These findings suggested that CTRP9 may exert its atheroprotective properties by upregulating cholesterol efflux and decreasing foam cell formation, which is the key step in RCT. Consistent results were also observed in RAW 264.7-derived foam cells in our vitro study. LXRs, key sterol-sensitive transcription factors, are essential for regulating cholesterol homeostasis and lipogenesis. The activation of LXR inhibits the absorption of extracellular cholesterol, while it promotes the efflux of cholesterol from peripheral tissues (Phillips 2014). ABCA1 and ABCG1 expressions are transcriptionally regulated by multiple processes, including PKCα pathway (Liu et al. 2013), PKC-η/phospholipase D2 (PLD2) signaling pathway (Park et al. 2013), and activation of LXR in macrophages (Costet et al. 2000; Kennedy et al. 2005). We have shown in our study that CTRP9 upregulated LXR-α, ABCA1, and ABCG1expression both in vivo and in vitro. This suggests the regulation of CTRP9 on ABCA1 and ABCG1 in RCT is mediated via LXRα pathway.

AMPK is a central regulator of cellular energy homeostasis. Previous studies have shown that in AMI model or diet-induced obesity mice, overexpression of CTRP9 enhances AMPK activation, while the knockout of CTRP9 reduced AMPK activation (Kambara et al. 2015; Peterson et al. 2013; Wei et al. 2014). Administration of globular CTRP9 significantly reduces TNF-α and MCP-1 production via the AdipoR1/AMPK/NF-kB signaling pathway in RAW 264.7 macrophages (Kambara et al. 2015). The anti-contractile effect of CTRP9 in perivascular adipose tissues is mediated by activating the AMPK-eNOS pathway (Han et al. 2018). All these studies suggest that AMPK is a major modulator in CTRP9 signaling. Consistent with this, we demonstrate that CTRP9 upregulates AMPK mRNA and protein levels in RAW 264.7-derived foam cells. When AMPK is knocked down, the upregulation of ABCA1, ABCG1, and LXR-α by CTRP9 is blocked. Also, the promotion of cholesterol efflux by CTRP9 is also decreased. These results suggest an AMPK-dependent mechanism involved in the upregulation of cholesterol efflux by CTRP9. Taken together, our in vivo and in vitro studies indicate that CTRP9 may regulate reverse cholesterol transport via AMPK-LXR-α-ABCA1/ABCG1 pathway.

Although our results confirm the protective role of human CTRP9 in atherosclerosis development, some limitations of this study should be noted. First, the main target cells for exogenous CTRP9 are not characterized. Many cell types including macrophages, endothelial cells, and VSMCs can be modulated by CTRP9. (Zheng et al. 2011; Liu et al. 2017; Lei et al. 2021) Our in vitro study validates the modulation of macrophage cholesterol efflux by CTRP9. Further investigation should be done to corroborate the key cells that contribute to the effect of CTRP9 on atherosclerosis. Second, the receptor mediating the activity of CTRP9 during atherosclerosis is unclear. Finally, the pharmacokinetics and pharmacodynamics of human CTRP9 in *ApoE*^−/−^ mice need to be further determined.

In conclusion, our study shows that CTRP9 prevents atherosclerotic plaque formation and enhances RCT in a mouse model of atherosclerosis. CTRP9 has the ability to stimulate the expression of ABCA1/ABCG1, and LXR-α through activation of AMPK signaling, consequently accelerating cholesterol efflux from foam cells. Therefore, delivery of CTRP9 may provide therapeutic benefits in the treatment of atherosclerosis.

## Supplementary Information

Below is the link to the electronic supplementary material.

Supplementary file1 (DOC 5653 KB)

## References

[CR1] Alagarsamy J, Jaeschke A, Hui DY (2022) Apolipoprotein E in cardiometabolic and neurological health and diseases. Int J Mol Sci 23:9892 36077289 10.3390/ijms23179892 PMC9456500

[CR2] Bonamassa B, Moschetta A (2013) Atherosclerosis: lessons from LXR and the intestine. Trends Endocrinol Metab 24:120–128 23158108 10.1016/j.tem.2012.10.004

[CR3] Costet P, Luo Y, Wang N, Tall AR (2000) Sterol-dependent transactivation of theABC1 promoter by the liver X receptor/retinoid X receptor. J Biol Chem 275:28240–28245 10858438 10.1074/jbc.M003337200

[CR4] Dean M, Rzhetsky A, Allikmets R (2001) The human ATP-binding cassette (ABC) transporter superfamily. Genome Res 11:1156–1166 11435397 10.1101/gr.184901

[CR5] Fang H, Judd RL (2018) Adiponectin regulation and function. Compr Physiol 8:1031–1063 29978896 10.1002/cphy.c170046

[CR6] Han F, Zhang Y, Shao M, Mu Q, Jiao X, Hou N, Sun X (2018) C1q/TNF‐related protein 9 improves the anti‐contractile effects of perivascular adipose tissue via the AMPK‐eNOS pathway in diet‐induced obese mice. Clin Exp Pharmacol Physiol 45:50–57 28902432 10.1111/1440-1681.12851

[CR7] Hong C, Tontonoz P (2014) Liver X receptors in lipid metabolism: opportunities for drug discovery. Nat Rev Drug Discov 13:433–444 24833295 10.1038/nrd4280

[CR8] Huang C, Zhang P, Li T, Li J, Liu T, Zuo A, Chen J, Guo Y (2019) Overexpression of CTRP9 attenuates the development of atherosclerosis in apolipoprotein E-deficient mice. Mol Cell Biochem 455:99–108 30426302 10.1007/s11010-018-3473-y

[CR9] Jung CH, Lee MJ, Kang YM, Lee YL, Seol SM, Yoon HK, Kang SW, Lee WJ, Park JY (2016) C1q/TNF-related protein-9 inhibits cytokine-induced vascular inflammation and leukocyte adhesiveness via AMP-activated protein kinase activation in endothelial cells. Mol Cell Endocrinol 419:235–243 26523509 10.1016/j.mce.2015.10.023

[CR10] Kambara T, Shibata R, Ohashi K, Matsuo K, Hiramatsu-Ito M, Enomoto T, Yuasa D, Ito M, Hayakawa S, Ogawa H, Aprahamian T, Walsh K, Murohara T, Ouchi N (2015) C1q/tumor necrosis factor-related protein 9 protects against acute myocardial injury through an adiponectin receptor I-AMPK-dependent mechanism. Mol Cell Biol 35:2173–2185 25870106 10.1128/MCB.01518-14 PMC4438248

[CR11] Kennedy MA, Barrera GC, Nakamura K, Baldan A, Tarr P, Fishbein MC, Frank J, Francone OL, Edwards PA (2005) ABCG1 has a critical role in mediating cholesterol efflux to HDL and preventing cellular lipid accumulation. Cell Metab 1:121–131 16054053 10.1016/j.cmet.2005.01.002

[CR12] Lei S, Chen J, Song C, Li J, Zuo A, Xu D, Li T, Guo Y (2021) CTRP9 alleviates foam cells apoptosis by enhancing cholesterol efflux. Mol Cell Endocrinol 522:111138 33352225 10.1016/j.mce.2020.111138

[CR13] Liang B, Wang X, Song X, Bai R, Yang H, Yang Z, Xiao C, Bian Y (2017) MicroRNA-20a/b regulates cholesterol efflux through post-transcriptional repression of ATP-binding cassette transporter A1Bian. Biochim Biophys Acta Mol Cell Biol Lipids 1862:929–938 28602962 10.1016/j.bbalip.2017.06.002

[CR14] Liu XY, Lu Q, Ouyang XP, Tang SL, Zhao GJ, Lv YC, He PP, Kuang HJ, Tang YY, Fu Y, Zhang DW, Tang CK (2013) Apelin-13 increases expression of ATP-binding cassette transporter A1 via activating protein kinase C α signaling in THP-1 macrophage-derived foam cells. Atherosclerosis 226:398–407 23290264 10.1016/j.atherosclerosis.2012.12.002

[CR15] Liu Q, Zhang H, Lin J, Zhang R, Chen S, Liu W, Sun M, Du W, Hou J, Yu B (2017) C1q/TNF‐related protein 9 inhibits the cholesterol‐induced vascular smooth muscle cell phenotype switch and cell dysfunction by activating AMP‐dependent kinase. J Cell Mol Med 21:2823–2836 28524645 10.1111/jcmm.13196 PMC5661105

[CR16] Liu Y, Wang X, Wang T, Zhang W, Chang Y, Dai W, Zhao J, Wang Z, Qi Y, Pan H (2022) J Healthc Eng 2022:635446 10.1155/2022/1635446 PMC8977318 35388328

[CR17] Maruyama S, Shibata R, Kikuchi R, Izumiya Y, Rokutanda T, Araki S, Kataoka Y, Ohashi K, Daida H, Kihara S, Ogawa H, Murohara T, Ouchi N (2012) Fat-derived factor omentin stimulates endothelial cell function and ischemia-induced revascularization via endothelial nitric oxide synthase-dependent mechanism. J Biol Chem 287:408–417 22081609 10.1074/jbc.M111.261818 PMC3249092

[CR18] Ouimet M, Barrett TJ, Fisher EA (2019) HDL and reverse cholesterol transport: basic mechanisms and their roles in vascular health and disease. Circ Res 124:1505–1518 31071007 10.1161/CIRCRESAHA.119.312617 PMC6813799

[CR19] Park DW, Lee HK, Lyu JH, Chin H, Kang SW, Kim YJ, Bae YS, Baek SH (2013) TLR2 stimulates ABCA1 expression via PKC-η and PLD2 pathway. Biochem Biophys Res Commun 430:933–937 23261454 10.1016/j.bbrc.2012.11.135

[CR20] Peterson JM, Wei Z, Seldin MM, Byerly MS, Aja S, Wong GW (2013) CTRP9 transgenic mice are protected from diet-induced obesity and metabolic dysfunction. Am J Physiol Regul Integr Comp Physiol 305:R522-533 23842676 10.1152/ajpregu.00110.2013 PMC3763026

[CR21] Phillips MC (2014) Molecular mechanisms of cellular cholesterol efflux. J Biol Chem 289:24020–24029 25074931 10.1074/jbc.R114.583658 PMC4148835

[CR22] Shibata R, Ohashi K, Murohara T, Ouchi N (2014) The potential of adipokines as therapeutic agents for cardiovascular disease. Cytokine Growth Factor Rev 25:483–487 25066649 10.1016/j.cytogfr.2014.07.005

[CR23] Sun Y, Yi W, Yuan Y, Lau WB, Yi D, Wang X, Wang Y, Su H, Wang X, Gao E, Koch WJ, Ma XL (2013) C1q/tumor necrosis factor–related protein-9, a novel adipocyte-derived cytokine, attenuates adverse remodeling in the ischemic mouse heart via protein kinase A activation. Circulation 128:S113-120 24030394 10.1161/CIRCULATIONAHA.112.000010 PMC3824619

[CR24] Sun H, Zhu X, Zhou Y, Cai W, Qiu L (2017) Hypaphorine attenuates lipopolysaccharide-induced endothelial inflammation via regulation of TLR4 and PPAR-γ dependent on PI3K/Akt/mTOR signal pathway. Int J Mol Sci 18:844 28420166 10.3390/ijms18040844 PMC5412428

[CR25] Tarling EJ, Edwards PA (2011) ATP binding cassette transporter G1 (ABCG1) is an intracellular sterol transporter. Proc Natl Acad Sci USA 108:19719–19724 22095132 10.1073/pnas.1113021108 PMC3241749

[CR26] Wang B, Tontonoz P (2018) Liver X receptors in lipid signalling and membrane homeostasis. Nat Rev Endocrinol 14:452–463 29904174 10.1038/s41574-018-0037-x PMC6433546

[CR27] Wang J, Hang T, Cheng XM, Li DM, Zhang QG, Wang LJ, Peng YP, Gong JB (2015) Associations of C1q/TNF-related protein-9 levels in serum and epicardial adipose tissue with coronary atherosclerosis in humans. Biomed Res Int 2015:971683 26457306 10.1155/2015/971683 PMC4589613

[CR28] Wei Z, Lei X, Petersen PS, Aja S, Wong GW (2014) Targeted deletion of C1q/TNF-related protein 9 increases food intake, decreases insulin sensitivity, and promotes hepatic steatosis in mice. Am J Physiol Endocrinol Metab 306:E779-790 24473438 10.1152/ajpendo.00593.2013 PMC3962615

[CR29] Westerterp M, Bochem AE, Yvan-Charvet L, Murphy AJ, Wang N, Tall AR (2014) ATP-Binding Cassette Transporters, Atherosclerosis, and Inflammation. Circ Res 114:157–170 24385509 10.1161/CIRCRESAHA.114.300738

[CR30] Wong GW, Krawczyk SA, Kitidis-Mitrokostas C, Ge G, Spooner E, Hug C, Gimeno R, Lodish HF (2009) Identification and characterization of CTRP9, a novel secreted glycoprotein, from adipose tissue that reduces serum glucose in mice and forms heterotrimers with adiponectin. FASEB J 23:241–258 18787108 10.1096/fj.08-114991 PMC2626616

[CR31] Yang Y, Li Y, Ma Z, Jiang S, Fan C, Hu W, Wang D, Di S, Sun Y, Yi W (2016) A brief glimpse at CTRP3 and CTRP9 in lipid metabolism and cardiovascular protection. Prog Lipid Res 64:170–177 27743997 10.1016/j.plipres.2016.10.001

[CR32] Yu XH, Fu YC, Zhang DW, Yin K, Tang CK (2013) Foam cells in atherosclerosis. Clin Chim Acta 424:245–252 23782937 10.1016/j.cca.2013.06.006

[CR33] Yu XH, Zhang DW, Zheng XL, Tang CK (2018) C1q tumor necrosis factor-related protein 9 in atherosclerosis: Mechanistic insights and therapeutic potential. Atherosclerosis 276:109–116 30056359 10.1016/j.atherosclerosis.2018.07.022

[CR34] Zhang Y, Zanotti I, Reilly MP, Glick JM, Rothblat GH, Rader DJ (2003) Overexpression of apolipoprotein A-I promotes reverse transport of cholesterol from macrophages to feces in vivo. Circulation 108:661–663 12900335 10.1161/01.CIR.0000086981.09834.E0

[CR35] Zhang H, Gong X, Ni S, Wang Y, Zhu L, Ji N (2019) C1q/TNF-related protein-9 attenuates atherosclerosis through AMPK-NLRP3 inflammasome singling pathway. Int Immunopharmacol 77:105934 31727560 10.1016/j.intimp.2019.105934

[CR36] Zheng Q, Yuan Y, Yi W, Lau WB, Wang Y, Wang X, Sun Y, Lopez BL, Christopher TA, Peterson JM, Wong GW, Yu S, Yi D, Ma XL (2011) C1q/TNF-related proteins, a family of novel adipokines, induce vascular relaxation through the adiponectin receptor-1/AMPK/eNOS/Nitric oxide signaling pathway. Arterioscler Thromb Vasc Biol 31:2616–2623 21836066 10.1161/ATVBAHA.111.231050 PMC3197867

