# Supplementary figures and images for: C1q/Tumor Necrosis Factor-Related Protein-9 Enhances Macrophage Cholesterol Efflux and Improves Reverse Cholesterol Transport via AMPK Activation
Source: Biochem Genet. 2024 Apr 10;63(2):1620–34. doi: 10.1007/s10528-024-10761-1 (PMC11929689; doi:10.1007/s10528-024-10761-1)

**Original Western blots**

Figure 3


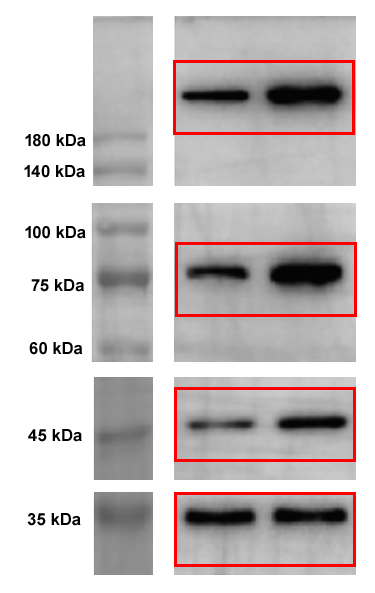


Figure 4


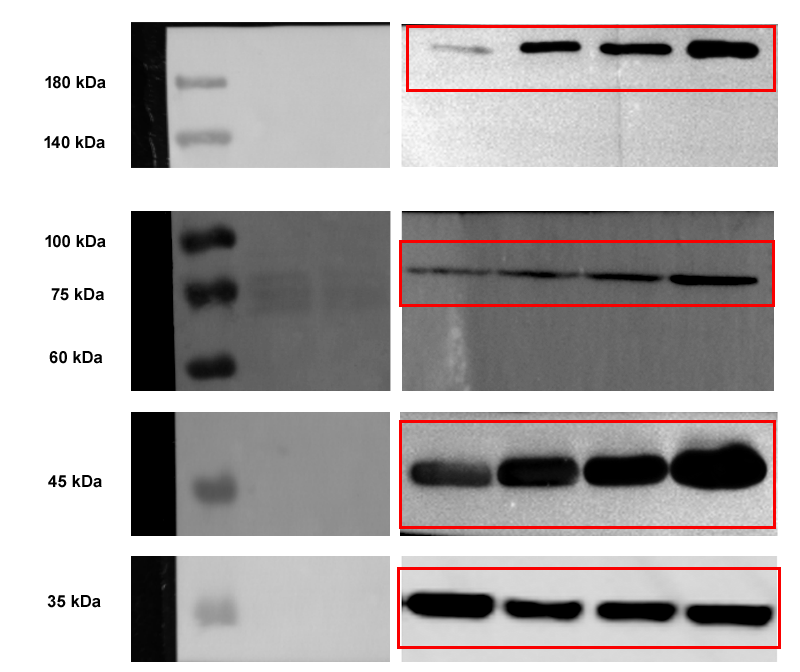


Figure 5


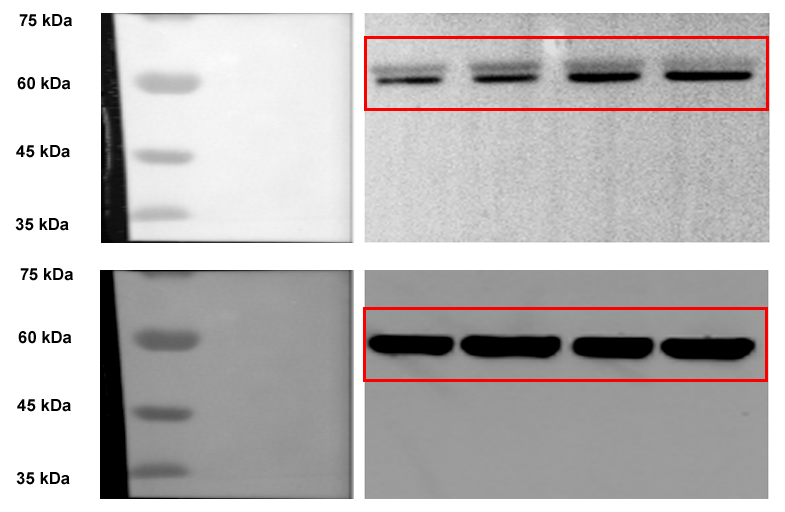


Figure 6


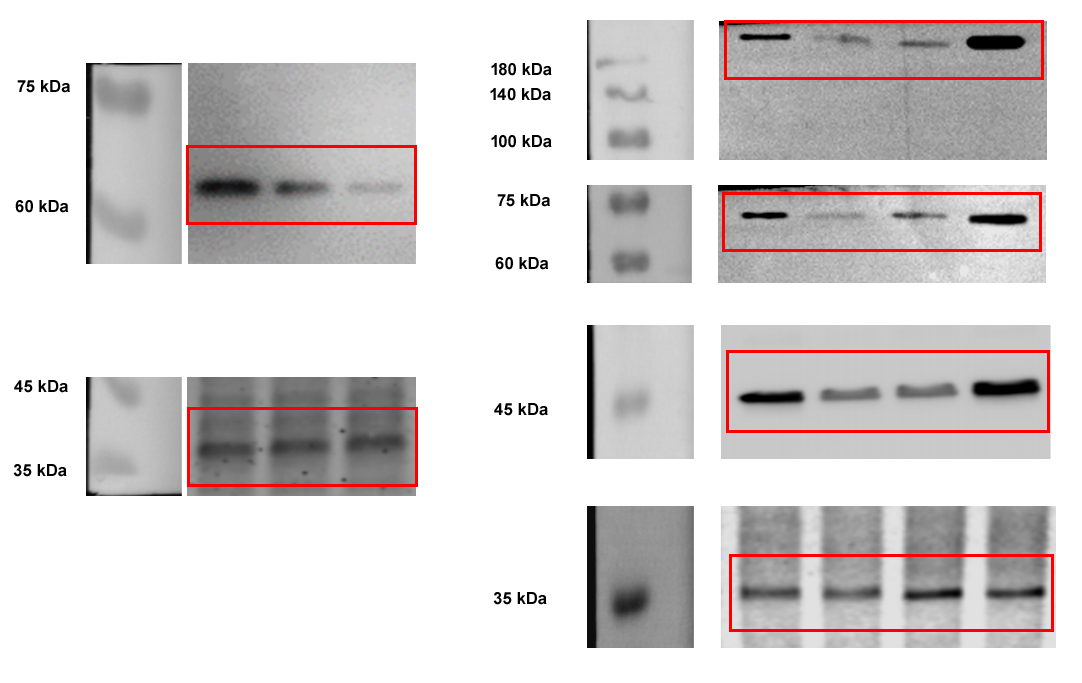

Supplement: Supplementary file 1 — Supplementary file1 (DOC 5653 KB) [file 10528_2024_10761_MOESM1_ESM.doc]
